# The Effect of Iron, Zinc, and Calcium Trunk and Root Injections on Fruit Characteristics and Leaf Mineral Content in “Mazafati” Date Palm

**DOI:** 10.1002/fsn3.71966

**Published:** 2026-05-31

**Authors:** Bahareh Damankeshan, Bahman Panahi, Ali Salehi Sardoei, Mansour Ghorbanpour

**Affiliations:** ^1^ Kerman Agricultural Research Education and Natural Resources Center, AREEO Kerman Iran; ^2^ Horticultural Sciences Research Institute, AREEO Karaj Iran; ^3^ Crop and Horticultural Science Research Department South Kerman Agricultural and Natural Resources Research and Education Center, AREEO Jiroft Iran; ^4^ Department of Medicinal Plants, Faculty of Agriculture and Natural Resources Arak University Arak Iran

**Keywords:** date palm, fruit traits, micronutrients, *Phoenix dactylifera*, trunk injection

## Abstract

This study evaluated the effects of trunk and root injections of iron (Fe), zinc (Zn), and calcium (Ca) on fruit characteristics and leaf mineral composition of “Mazafati” date palm ( *Phoenix dactylifera* L. cv. “Mazafati”) under orchard conditions in Kerman Province, Iran. A randomized complete block design with nine treatments was applied to 15‐year‐old palms: trunk injection of ferrous sulfate (25, 75, 150 g L^−1^), root injection of ferrous sulfate (75 g L^−1^), trunk injection of zinc aminochelate (2, 4 g L^−1^), trunk injection of calcium aminochelate (3, 5 mL L^−1^), and an untreated control (*n* = 3 replications). Fruit physical traits and leaf mineral concentrations were measured at the rutab stage and ~100 days post‐injection, respectively. Data were analyzed using ANOVA, Pearson correlation, and stepwise regression. Trunk injection of Fe at 150 g L^−1^ maximized flesh weight (16.38 g) and seed weight (1.31 g), while Zn at 4 g L^−1^ yielded the highest total fruit weight (17.59 g). Fe injection significantly increased leaf Fe, Mg, and Ca concentrations; Zn injection elevated leaf Zn and K but reduced Fe and Mg. Pearson correlations revealed positive Fe–Mg (*r* = 0.42) and Fe–Ca (*r* = 0.38) associations, and negative Zn–Mg (*r* = −0.41) and Zn–Fe (*r* = −0.30) interactions. Stepwise regression identified fruit flesh weight and leaf P as the strongest predictors of fruit weight (*R* ^2^ = 82.1%, *p* ≤ 0.01). Trunk injection of Fe (150 g L^−1^) and Zn (4 g L^−1^) at optimized dosages significantly improved key fruit traits and leaf mineral status in “Mazafati” date palm. However, observed nutrient antagonisms (e.g., Zn–Fe, Zn–Mg) underscore the need for balanced fertilization strategies. This approach shows promise for enhancing productivity in calcareous soils where conventional uptake is limited, though multi‐season trials and economic analyses are recommended before wide‐scale adoption.

## Graphical abstract

This study, conducted to evaluate the effects of trunk and root injections of iron (Fe), zinc (Zn), and calcium (Ca) on fruit characteristics and leaf mineral composition of “Mazafati” date palm ( *Phoenix dactylifera* L. cv. “Mazafati”), yielded several important findings. Trunk injection of iron and zinc significantly improved fruit weight and flesh content in “Mazafati” date palm. Fe injection increased leaf Fe, Mg, and Ca levels, whereas Zn injection raised Zn and K but reduced Fe and Mg, revealing antagonistic nutrient interactions. The study concludes that trunk injection of micronutrients at optimized doses is a promising strategy for enhancing date palm productivity, particularly in calcareous soils, provided that balanced fertilization practices are applied to avoid secondary deficiencies.

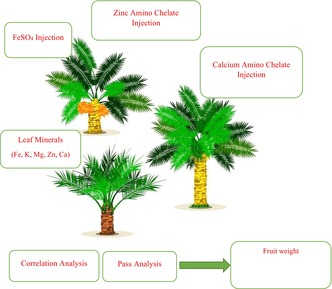

## 1 Introduction

Iran, producing an average of 1,283,499 tons of dates annually (13.58% of global production), ranks among the world's leading date producers (Food and Agriculture Organization of the United 2020). Among various cultivars, the “Mazafati” date palm ( *Phoenix dactylifera* L. cv. “Mazafati”), cultivated on approximately 20,000 ha, occupies the largest share in Kerman Province (Damankeshan et al. 2022) and is exported to over 27 countries (Rafiee et al. 2022). Owing to its high economic value, this cultivar has consistently attracted research attention to enhance yield and improve fruit quality (Panahi et al. 2023).

Soil fertilization is a common practice for improving plant nutrition (Namaswa et al. 2025), and its integration with site preparation techniques such as soil plowing can further enhance nutrient availability and biomass productivity in plant ecosystems (Varnagirytė‐Kabašinskienė and Survila 2025); however, conventional chemical fertilizers often exhibit low efficiency, with less than 20% uptake by plants, while the remainder can contribute to water and soil contamination (Sharma et al. 2021). The application of organic fertilizers and amino acid–chelated micronutrients, particularly under nutrient‐deficient conditions, has been reported to promote growth and increase both macro‐ and micronutrient contents (Kurdy and Al‐Dulaimy 2023). In “Mazafati” date palms, foliar spraying of aminochelates has shown positive quantitative and qualitative effects (Panahi et al. 2023). Previous studies have indicated that iron chelate enhances total soluble solids, reducing sugars, and fruit size (Faisal et al. 2018); calcium chelate improves vegetative growth (Jasim et al. 2016); and Fe and Zn aminochelates alleviate yield losses under salinity stress (Sh Sadak et al. 2015).

Nutrient application methods include soil application, foliar spraying, and trunk injection. Trunk injection is recognized as the most efficient approach for directly delivering compounds into the xylem, thereby overcoming limitations of penetration and evaporation (Archer et al. 2022; Doccola and Wild 2012). Beyond nutrient delivery, trunk injection is employed in pest and disease management, including as a biological control method (Berger et al. 2015; Sutanto et al. 2023; Hu et al. 2026), and reduces environmental risks by limiting pesticide dispersion (Berger and Laurent 2019; Gyuris et al. 2024). In “Mazafati” date palms, trunk injection with salicylic acid has been reported to mitigate cluster desiccation damage (Damankeshan et al. 2022), while trunk injection of Fe or Zn has been shown to improve yield (Nadia et al. 2017).

Although several studies have documented the benefits of nutrient injection in date palms, no comparative assessment has been carried out on the effects of trunk and root injections of Fe, Zn, and Ca on leaf mineral composition and fruit traits of the “Mazafati” cultivar. Considering the critical role of nutrition in enhancing date palm productivity and the hypothesis that nutrient absorption is higher via injection, the present study was conducted to evaluate the effects of ferrous sulfate, zinc aminochelate, and calcium aminochelate injections on quantitative and qualitative fruit parameters and leaf mineral concentrations of “Mazafati” date palms under orchard conditions in Kerman Province, southeastern Iran.

## 2 Materials and Methods

### 2.1 Plant Materials and Experimental Set Up

**FIGURE 1:**
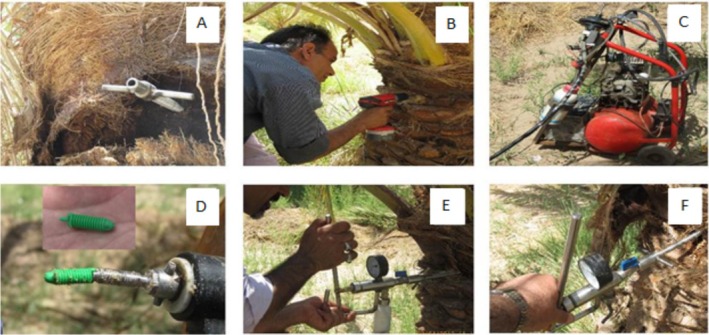
Stages of nutrient injection in the trunks of date palms. Chemical solution injection pump device for fruit trees (A), drill creating a hole in the tree trunk (B), installation of the injection device on the tree trunk (C), solution injection (D and E), covering the hole with a plastic cap (F).

This study was conducted on fifteen‐year‐old “Mazafati” date palms ( *Phoenix dactylifera* L. cv. “Mazafati”) at the Azizabad–Bam Agricultural Research Station, located at an elevation of 741 m above sea level, with geographical coordinates 28°56′ N and 58°45′ E. The experiment was laid out as a randomized complete block design (RCBD) with nine injection treatments applied to date palm trees: trunk injection of ferrous sulfate heptahydrate (FeSO_4_·7H_2_O) at concentrations of 25, 75, and 150 g L^−1^; root injection of FeSO_4_·7H_2_O at 75 g L^−1^. Root injection of FeSO_4_·7H_2_O was performed only at 75 g L^−1^, which was selected as an intermediate concentration to allow a practical comparison between root and trunk injection methods and to avoid potential adverse effects associated with high concentrations of micronutrients in the root zone (Marschner 1995); trunk injection of zinc aminochelate at 2 and 4 g L^−1^; trunk injection of calcium aminochelate at 3 and 5 mL L^−1^; and an untreated control. Each treatment was replicated three times, for a total of 27 trees (Table 1). Considering the greater cost‐effectiveness of FeSO_4_·7H_2_O for growers, this compound was selected for Fe supply, while Zn and Ca were provided in the form of aminochelates owing to their superior absorption efficiency. Treatments were administered at the end of the *kimri* stage, below the lowest whorl of leaves, approximately 130 cm above ground level. Injections were performed using an injection pump (Figure 1a) through a hole drilled into the trunk with a hand auger (Figure 1b). The injector screw was inserted into the drilled hole (Figure 1c), and injection was then carried out (Figure 1d,e). In this method, compound uptake occurs via the plant's natural xylem sap flow driven by transpiration. Although nutrient translocation by this method is somewhat slower than with other injection techniques (Archer et al. 2022), it causes the least damage to the tree. The fertilizer solution was prepared in 1 L of water and injected into four holes (diameter: 10 mm; depth: 10–12 cm) at a pressure below 15 psi. For injection treatments, this solution was administered by injecting 80 mL into each of the four drilled holes, resulting in a total applied volume of 320 mL per tree. The injection process was deliberately carried out slowly at each injection site to prevent leakage or localized phytotoxicity. After nutrient injection, the holes were disinfected with 0.1% potassium (K) permanganate (KMnO_4_), and the wound was sealed with a plastic plug using an automatic screwdriver (Figure 1f). For the FeSO_4_.7H_2_O root injection treatment, the procedure was similar to trunk injection, except that selected roots, located approximately 30 cm from the palm trunk and buried at a depth of about 15 cm, with diameters of 25–30 mm, were used for drilling and injection. Standard orchard management practices including pruning, pollination, proper cluster thinning, control of red spider mite (*Tetranychus urticae*), and cluster bagging were uniformly applied to all experimental trees. One month after pollination, six healthy, pollinated clusters from four directions around each tree were selected for data recording, and all other clusters were removed at the *kimri* stage.

**TABLE 1:** Description of experimental treatments.

Treatment	Description of experimental	Injection site
F25	FeSO_4_ (7H_2_O): 25 g/L	Trunk
F75	FeSO_4_ (7H_2_O): 75 g/L	Trunk
F150	FeSO_4_ (7H_2_O): 150 g/L	Trunk
F75	FeSO_4_ (7H_2_O): 75 g/L	Root
Zn2	Zink Amino Chelate: 2 g/L	Trunk
Zn4	Zink Amino Chelate: 4 g/L	Trunk
Ca3	Calcium Amino Chelate: 3 mL/L	Trunk
Ca5	Calcium Amino Chelate: 5 mL/L	Trunk
Control	Without injection	Trunk

### 2.2 Measured Parameters

Two categories of measurements were carried out: fruit physical parameters and leaf mineral concentrations.

### 2.3 Fruit Physical Parameters

From a total of six clusters per tree, 60 fruits were randomly harvested at the rutab stage in the experimental orchard. In the laboratory, 30 fruits per tree were randomly selected to evaluate quantitative fruit traits. The recorded parameters included fruit length, diameter, weight, pulp weight, seed weight, seed length, and seed diameter. Fruit and seed weights were determined using a digital balance (accuracy: 0.001 g), and measurements of fruit and seed length and diameter were taken using a caliper.

### 2.4 Leaf Mineral Concentration

Approximately 100 days after injection, 20 leaflet samples were collected from the middle section of the second frond from each experimental tree and transported to the laboratory (Mohabi and Nabhani 2013). Leaf samples were washed with deionized water and oven‐dried for 24 h at 70°C (Memmert GmbH, Germany). A 0.5 g subsample of the powdered material was ashed in a muffle furnace at 500°C for 4 h. The resultant ash was digested with 5 mL of 2 N hydrochloric acid, and the digest was diluted to 50 mL with deionized water. This extract was analyzed to determine K, sodium (Na), phosphorus (P), Ca, Fe, manganese (Mn), copper (Cu), Zn, boron (B), and chloride (Cl) concentrations in palm leaves. Micronutrients (Fe, Cu, Mn, and Zn) were extracted using the diethylene triamine pentaacetic acid (DTPA) method (Lindsay and Norvell 1978) and quantified by atomic absorption spectrophotometry.

#### 2.4.1 Sodium and Potassium Content

Na and K concentrations in date palm leaves were determined by flame photometry using a Jenway PEP7 flame photometer (Germany) following the method of Isaac and Kerber (1971).

#### 2.4.2 Phosphorus Content

P was measured using the ammonium molybdate ammonium vanadate (yellow) method. Briefly, 5 mL of the extract obtained in the previous step was mixed with 10 mL of ammonium molybdate ammonium vanadate reagent and diluted to 50 mL with deionized water (Noonan and Holcombe 1975). Absorbance was read at a wavelength of 470 nm using a T60 UV/Vis spectrophotometer (PG Instruments Ltd., UK).

#### 2.4.3 Calcium Content

Ca concentration was determined by titration according to (Klute 1986). For analysis, 5 mL of the prepared extract was transferred to a 50 mL Erlenmeyer flask and diluted to 25 mL with deionized water. Subsequently, 5 drops of 4 N sodium hydroxide (NaOH) were added, followed by 0.5 g of a dry indicator mixture composed of ammonium purpurate (murexide) and potassium sulfate (prepared by mixing 0.5 g murexide with 100 g potassium sulfate). The mixture was stirred thoroughly until a red‐orange color appeared. Titration was performed using EDTA solution (prepared by dissolving 1.86 g EDTA‐Na and 0.5 g OMgCl_2_H_2_ in 1 L of deionized water) until the color of the solution shifted from red‐orange to violet. Ca concentration was then calculated using the standard formula.

$$\mathrm{Ca}\;\left(\mathrm{mg}/L\right)=V_{\text{sample}}\times V_{\text{EDTA}}\;\left(\mathrm{Ca}\right)\times N_{\text{EDTA}}\times W_{\mathrm{Ca}}$$

In the titration formula, *V*_sample_ refers to the sample volume (mL), *V*_EDTA_ is the volume of EDTA solution used in the titration (mL), *N*_EDTA_ is the normality of the EDTA solution (meq L^−1^), and *W*_Ca_ is the equivalent weight of calcium (mg meq^−1^), which is typically 20.04 mg meq^−1^. This value is calculated based on the molecular weight and valence of calcium.

#### 2.4.4 Magnesium Content

Mg concentration was determined using a complexometric EDTA titration method following Ryan et al. (2001), an approach that has been widely applied in recent plant nutrition studies (e.g., Panhwar et al. 2020; Fu et al. 2024). Briefly, 5 mL of the extract was diluted to 25 mL with deionized water. Four drops of Eriochrome Black T indicator and 10 drops of ammonium chloride buffer were added, and the solution was titrated with EDTA until the color changed from brick red to blue or green. Calcium was determined separately as described above, and magnesium concentration was calculated by difference from the total Ca + Mg titration value.

$$\mathrm{Mg}\;\left(\mathrm{mg}/L\right)=V_{\text{EDTA}}\;\left(\mathrm{Mg}+\mathrm{Ca}\right)-V_{\text{EDTA}}\;\left(\mathrm{Ca}\right)\times F$$

$$F\;\left(\mathrm{mg}/L\right)=\frac{W_{\mathrm{Mg}}}{N_{\text{EDTA}}}$$

*V*_EDTA_ (Mg + Ca) represents the volume of EDTA used for titrating the mixture of magnesium and calcium (mL), and *V*_EDTA_ (Ca) represents the volume of EDTA used exclusively for titrating calcium (mL). *F* is the EDTA conversion factor, *W*_Mg_ is the equivalent weight of magnesium (mg meq^−1^), and *N*_EDTA_ denotes the normality of the EDTA solution (meq L^−1^).

#### 2.4.5 Nitrogen Determination

Total nitrogen (N) content was determined using the Kjeldahl method following AOAC Official Methods of Analysis (AOAC 2023). A 0.20 g portion of the date palm leaf sample was weighed with an accuracy of 0.0001 g, wrapped in paper, and placed into digestion tubes for 3 h. Each sample received one Cupric (copper‐selenium) catalyst tablet, 2 mL of deionized water, and 10 mL of concentrated sulfuric acid (H_2_SO_4_). Following complete digestion, the samples were cooled for 1 h and then transferred to the distillation apparatus. At this stage, a few drops of a color indicator were added, turning the solution red. A 4% boric acid solution, 40% Na hydroxide (NaOH), and deionized water were used during distillation. N released from the samples as vapor was absorbed into an Erlenmeyer flask containing the indicator solution, resulting in a color change from red to green, finally yielding a clear green solution. Subsequently, the distillate was titrated using a digital burette containing 0.1 N hydrochloric acid (HCl) until the indicator changed from light green to red. The burette reading was recorded immediately, and leaf N content was calculated using the standard Kjeldahl formula.

$$T.N\;\left(\%\right)=\frac{\left(\mathrm{HCl}\times \text{NHCl}\times 100\times 14\right)}{\left(W\times 1000\right)}$$

In this equation: T.N represents the percentage of total nitrogen in the sample; HCl is the volume of hydrochloric acid (mL) used for titration; NHCl is the normality of the hydrochloric acid (0.1 N); *W* is the sample weight in grams; the constant 14 denotes the atomic weight of nitrogen (mg meq^−1^); 100 is used to convert the value to a percentage; and 1000 converts the sample weight from grams to milligrams.

### 2.5 Statistical Analysis

Statistical analyses included descriptive statistics, Duncan's multiple range test (DMRT) at *p* ≤ 0.05, Pearson's correlation coefficient, and stepwise regression analysis at *p* < 0.01, all performed using SPSS software (version 26; IBM Corp., Armonk, NY, USA). Path analysis, used to determine direct and indirect effects, was performed using AMOS software (version 24) to generate causal diagrams.

## 3 Results

### 3.1 Effects of Nutrient Injection Treatments on Fruit Physical Characteristics

**FIGURE 2:**
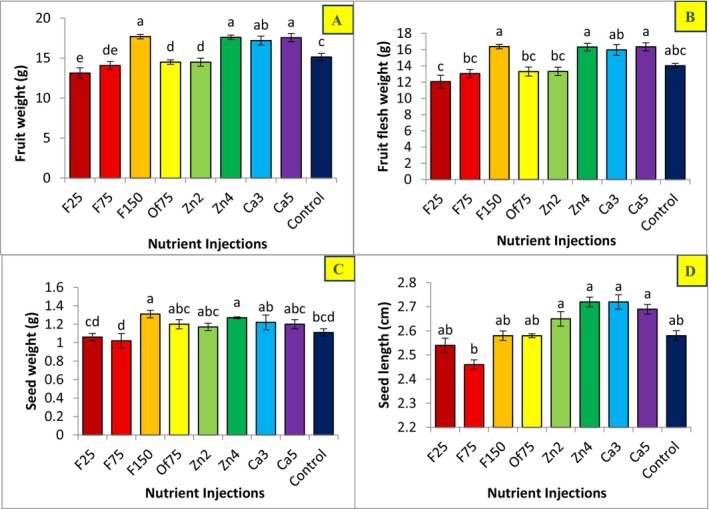
Effect of treatment levels on the fruit weight (A), fruit flesh weight (B), seed weight (C), and seed length (D) of Mazafati date palm ( *P. dactylifera* cv. Mazafati). In each figure, means with same letters had non‐significant difference with each other (*p* < 0.05).

Analysis of fruit physical characteristics—including fruit weight, flesh weight, seed weight, and seed length—revealed differential responses to the applied nutrient injection treatments. Statistical comparison of mean values highlighted distinct treatment effects (Figure 2).

The highest flesh weight (16.38 g) and seed weight (1.31 g) were recorded in the F150 treatment (injection of 150 g L^−1^ Fe). The highest fruit weight (combined flesh and seed), with a mean of 17.69 g, was also observed in F150; however, this value did not differ statistically from those of the two other treatments. The Zn4 (injection of 4 g L^−1^ Zn) and Ca5 (injection of 5 mL L^−1^ Ca) treatments yielded fruit weights of 17.59 and 17.55 g, respectively—values closest to F150—though the effect of Fe appeared more pronounced in the mean comparisons.

Zn4 and Ca5 treatments produced the greatest seed length in “Mazafati” date fruits, whereas the F75 treatment (injection of 75 g L^−1^ Fe) resulted in the lowest seed length and seed weight. Lower or intermediate Fe levels (F25 and F75) constrained seed development, while the higher Fe level (F150) exerted a more positive influence on overall fruit weight.

Although the Ca5, F150, and Zn4 treatments produced higher fruit weights relative to the control, these increases were not statistically significant (*p* > 0.05), indicating that while the treatments contributed to fruit weight improvement, the magnitude of change did not reach statistical significance compared to the untreated control. Notably, only the F25 (injection of 25 g L^−1^ Fe) and F75 treatments resulted in a statistically significant reduction in fruit weight compared with the control (*p* < 0.05).

### 3.2 Effects of Injection Treatments on Leaf Mineral Composition

**FIGURE 3:**
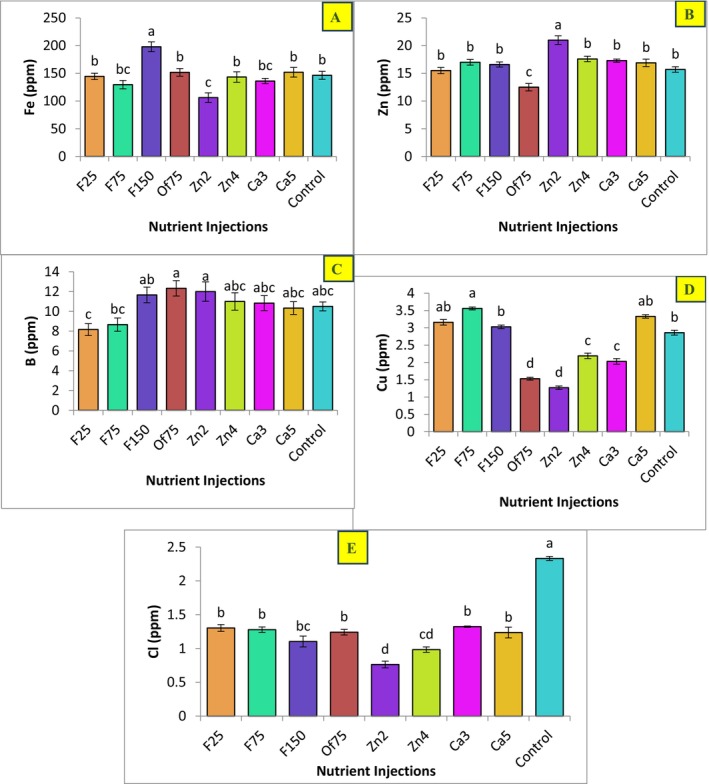
Effect of applied treatment levels on the concentration of iron (A), zinc (B), boron (C), copper (D), and chlorine (E) in the leaves of Mazafati date palm ( *P. dactylifera* cv. Mazafati). In each figure means with same letters had no significant difference with each other (*p* < 0.05).

In this study, Fe injection influenced the concentrations of multiple mineral elements in the leaves of “Mazafati” date palms. Specifically, the F150 treatment (injection of 150 g L^−1^ Fe) significantly increased leaf concentrations of Mg, Ca, and Fe, whereas the F75 treatment (injection of 75 g L^−1^ Fe) resulted in a significant increase in Cu concentration (Figure 3).

A dose‐dependent response was observed for K and Ca: as the injected Fe concentration increased from 25 to 150 ppm, leaf concentrations of K and Ca exhibited an upward trend, with the effect becoming more pronounced at the higher Fe level (F150).

For boron (B), the F25 and F75 treatments significantly reduced leaf B concentration relative to the control. In contrast, increasing the Fe dose to 150 ppm (F150) reversed this trend, leading to a significant increase in leaf B concentration compared with both the control and the lower Fe treatments.

**FIGURE 4:**
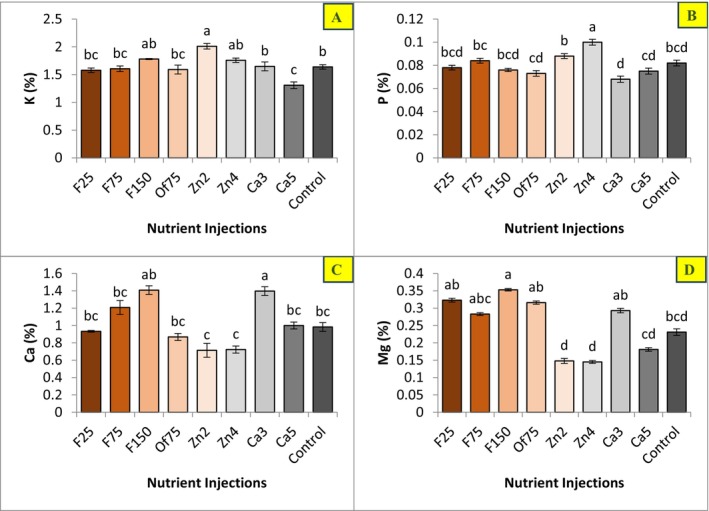
Effect of applied treatment levels on the concentration of potassium (A), phosphorus (B), calcium (C), and magnesium (D) in the leaves of Mazafati date palm ( *P. dactylifera* cv. Mazafati). In each figure, means with same letters had no significant difference with each other (*p* < 0.05).

In this study, Zn injection (Zn2; injection of 2 g L^−1^ Zn) significantly increased leaf Zn and K concentrations and significantly decreased leaf Fe, Mg, Cu, and Ca concentrations (Figures 3 and 4). The highest leaf Zn concentration was observed in the Zn2 treatment, whereas the lowest Zn concentration was recorded in plants receiving the Fe75 root injection treatment (injection of 75 g L^−1^ Fe).

Contrary to some previous studies, no clear dose‐dependent increase in leaf Zn concentration was observed with higher levels of injected Zn in this study; rather, the highest leaf Zn concentration corresponded to the Zn2 treatment. These findings suggest that identifying an appropriate Zn concentration—rather than maximizing application rate—may optimize plant performance.

Additionally, the highest leaf P concentration was recorded in Zn treatments, a response that coincided with increased fruit weight. The highest leaf K concentration was also obtained in the Zn2 treatment. In contrast, leaf K concentration decreased under Ca treatments (Ca5; injection of 5 mL L^−1^ Ca) (Figure 4).

Ca treatments resulted in a significant increase in leaf Ca levels and a significant decrease in leaf P levels (Figure 4). Specifically, at the lower Ca concentration (Ca3 treatment; injection of 3 mL L^−1^ Ca), leaf Mg content also showed a significant increase compared with the control.

Notably, Ca treatments, similar to the effects observed with Fe and Zn treatments, increased date fruit weight in the examined samples. This finding indicates a positive and comparable effect of these micronutrients in improving product quality, as reflected by enhanced fruit weight.

### 3.3 Relationships Between Fruit Traits and Mineral Elements

**FIGURE 5:**
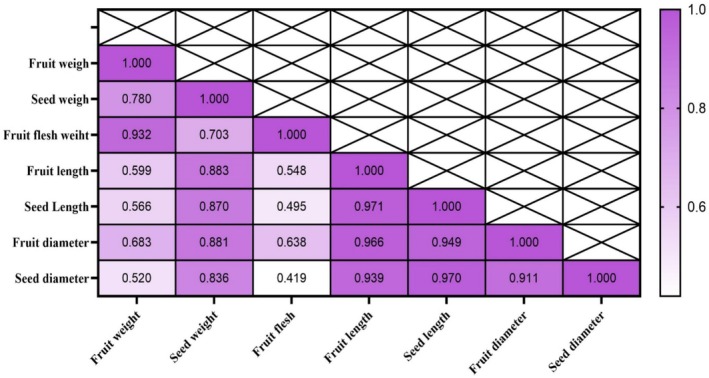
Correlation coefficients among morphological parameters of “Mazafati” date fruit ( *Phoenix dactylifera* L. cv. “Mazafati”). The correlation matrix illustrates the relationships between fruit weight, flesh weight, seed weight, seed length, and other measured morphological traits. Significant positive or negative correlations (*p* < 0.05) are indicated by color intensity or symbol size.

Correlation analysis of fruit morphological traits revealed that fruit weight—the most important trait assessed—exhibited the highest positive and significant correlations with flesh weight (*r* = 0.932), seed weight (*r* = 0.780), and fruit diameter (*r* = 0.683) (Figure 5).

**FIGURE 6:**
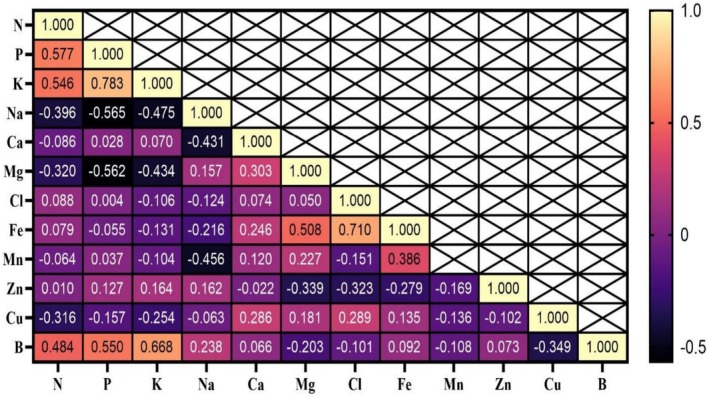
Correlation coefficients of leaf elements of Mazafati variety date ( *P. dactylifera* cv. Mazafati).

The correlations among leaf mineral elements are presented in Figure 6. According to these results, Fe showed a positive and significant correlation with Mg (*r* = 0.508, *p* < 0.01) and a significant antagonistic relationship with Zn (*r* = −0.279, *p* < 0.05). Zn, in addition to its negative association with Fe, exhibited significant negative correlations with Mg (*r* = −0.339, *p* < 0.05) and Cl (*r* = −0.323, *p* < 0.05). Furthermore, Ca showed positive and significant correlations with the divalent cations Mg (*r* = 0.303) and Cu (*r* = 0.286); significance levels for these Ca‐associated correlations are indicated in Figure 6.

**FIGURE 7:**
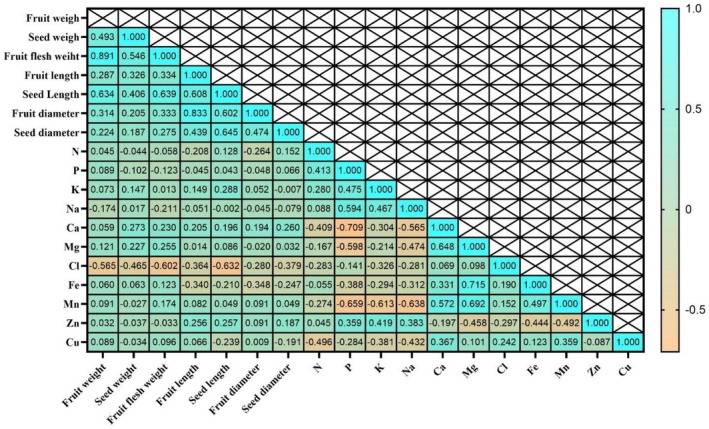
Heat map of mutual relations of variables in correlation coefficient for fruit indicators and elements nutrients.

The correlation matrix indicated strong interrelationships among most of the traits examined (Figure 7). The Cl^−^ ion showed the lowest correlations with fruit traits, and this relationship was negative and significant according to Duncan's test. In the correlation map, Cl^−^ exhibited very strong and negative correlations with certain fruit traits—for example, fruit weight (*r* = −0.565) and fruit flesh weight (*r* = −0.602). Negative and significant relationships were also observed between Fe and fruit length, seed length, fruit diameter, and seed diameter. These findings indicate that changes in one trait are associated with reciprocal increases or decreases in other correlated traits.

### 3.4 Path Analysis

To investigate the relationships among traits in greater detail, path analysis was performed. Path analysis partitions the relationships between independent and dependent variables into direct and indirect effects, thereby assisting researchers in accurately interpreting complex trait interactions. When high correlations exist among predictor variables, multicollinearity may occur; in conventional path analysis, multicollinearity can distort model estimates, leading to unstable parameter selection accompanied by inflated standard errors. Sequential path analysis is one method for mitigating multicollinearity and simplifying the complexity of relationships among traits, and this approach was applied in the present study.

The direct effects, representing the sum of the influences of the studied traits on fruit weight, are presented in Table 2. Results from the stepwise regression stage indicated that only two models were generated, and the traits fruit flesh weight and P had the greatest impact on determining fruit weight (Table 2). The coefficient of determination (*R* ^2^) was 78.6% for the first model and 82.1% for the second model, indicating that the inclusion of P as a secondary predictor improved the explanatory power of the model.

**TABLE 2:** Stepwise regression for fruit weight.

Variables entered to model	Step	
	1	2
Intercept	−1.328	−3.860
Fruit flesh weight	1.163	1.196
P	—	20.761
*R* ^2^ (%)	78.6	82.1

**FIGURE 8:**
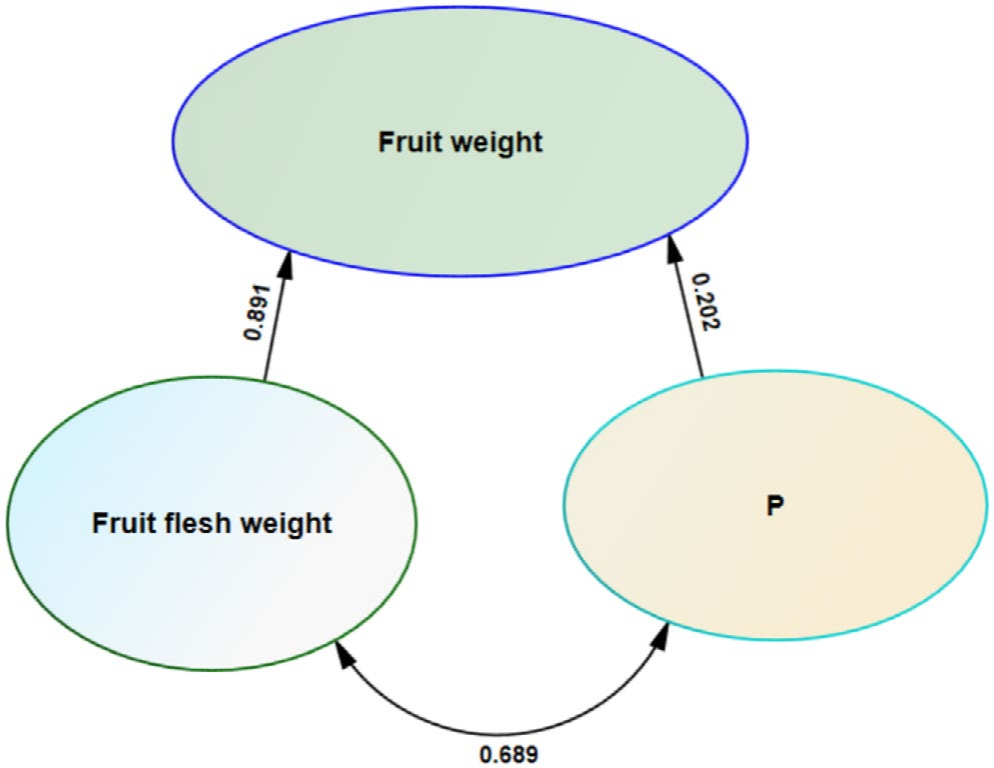
Path analysis of studied traits on fruit weight.

The highest positive direct effects on fruit weight were observed for fruit flesh weight (0.891) and P (0.202) (Table 3 and Figure 8). The greatest positive indirect effect on fruit weight occurred through fruit flesh weight (0.202) (Figure 8).

**TABLE 3:** Direct (diagonal values) and indirect (values outside diagonal) effects of studied traits on fruit weight.

Trait	Fruit flesh weight	P	Correlation with fruit weight
Fruit flesh weight	0.891	0.041	0.932
P	0.202	0.202	0.089

## 4 Discussion

The results of this study, conducted under actual orchard conditions using trunk injection, demonstrated that Zn application significantly influenced fruit growth traits and the leaf mineral composition of *Phoenix dactylifera* L. cv. “Mazafati”. This delivery method enabled more direct and efficient Zn uptake, and the findings obtained have high potential for generalization to commercial agricultural conditions. Specifically, the Zn_4_ treatment significantly increased total fruit weight and fruit flesh weight. This notable increase in fruit weight underscores the positive role of Zn in enhancing fruit yield and quality, aligning with the findings of other researchers. For example, Babu et al. (1984) and Kurdy and Al‐Dulaimy (2023) reported that Zn application improved quantitative and qualitative fruit traits, including fruit weight, diameter, and length, as well as seed size, seed weight, and bunch weight in date palm and other horticultural crops. Similarly, studies such as Hosseini Farahi et al. (2010) on grapes have shown that an appropriate Zn dose improves physiological efficiency and increases yield. Zn injection also led to a substantial increase in Zn concentration in leaf tissues, evidencing the effective uptake and translocation of this element within the plant following trunk injection. This elevated leaf Zn concentration may enhance the activity of several key enzymes in which Zn is structurally involved (Bhatla et al. 2018; Munawar et al. 2020). Zn acts as a cofactor in numerous essential metabolic processes, playing critical roles in the metabolism of proteins, starch, sugars, and auxin (Rout and Das 2009; Whiley et al. 1991). The improvement in fruit quality and quantity resulting from Zn application can be attributed to its effects on increasing the formation and translocation of carbohydrates and carbohydrate‐related enzymes, which are essential for fruit filling (Ramezani and Shekafandeh 2009). Moreover, Zn contributes to auxin synthesis, a key growth regulator that promotes cell expansion (Hosseini Farahi et al. 2010), and the observed increase in fruit weight may be linked to this mechanism. The enhancement of fruit weight following Zn treatment may further be attributed to zinc's role in fruit growth and developmental processes through its involvement in tryptophan metabolism and auxin biosynthesis (Jalal et al. 2024; Bhatla and Lal 2023).

An assessment of other elemental concentrations in leaves revealed complex interaction patterns in response to Zn treatments. The Zn_2_ treatment significantly increased leaf P and K concentrations. The observed increase in leaf P and K under Zn application may be supported by the central role of phosphorus in energy metabolism and ATP‐mediated biochemical processes, as well as the key function of potassium in photosynthate translocation and carbohydrate allocation to sink organs (Hawkesford et al. 2011; Zörb et al. 2014; Hasanuzzaman et al. 2018), consistent with classical descriptions by Mengel and Kirkby (1987). The increase in K under Zn influence may be attributed to stimulation of ATPase pumps and the consequent enhanced K uptake, as previously reported in maize (Karmollachaab and Gharineh 2013). Other studies have also reported that the simultaneous application of Zn and K has the greatest effect on increasing date palm fruit weight (Rusta 2012). However, in the present study, Zn application led to a significant reduction in the concentrations of elements such as Cu, Ca, and Mg in leaves, which may indicate an antagonistic interaction between Zn and these elements during uptake or translocation. This reduction in certain elements parallels the findings of Kolberg et al. (2022), who reported that elevated Zn concentrations can substantially decrease the uptake and accumulation of elements such as K and Mn in plant shoots. Such decreases may also be associated with altered plant water status, reflecting a direct influence of Zn on plant metabolism and physiology. These findings highlight the importance of precisely regulating Zn dosage and formulation in date palm nutrient management programs to maximize performance benefits while maintaining internal nutrient balance. A negative correlation between leaf Zn concentration and Fe accumulation was also observed. This antagonistic interaction may arise from competition between Zn and Fe for translocation via shared transporters in the root system or through iron‐absorbing phytosiderophores (Kolberg et al. 2022). The highest leaf Zn concentration was recorded for the Zn_2_ treatment, while the lowest was observed with FeSO_4_ root injection. Interestingly, contrary to previous reports suggesting that Zn uptake and translocation in leaves are proportional to the amount of Zn applied (Hosseini Farahi et al. 2010), such a relationship was not observed in the present study. Instead, increasing the injected Zn dose resulted in a decrease in leaf Zn concentration. This finding may reflect complexities in Zn uptake and translocation within the date palm system, or possibly saturation of uptake mechanisms at higher doses, and warrants further investigation to elucidate the underlying physiological phenomenon. Overall, the results of this study emphasize the importance of Zn as an essential micronutrient in improving the quantitative performance of *Phoenix dactylifera* cv. “Mazafati” through trunk injection under orchard conditions. While Zn exerted positive effects by increasing fruit weight and the concentration of certain elements (such as P and K), its interactions with other elements (including Cu, Ca, Mg, and Fe) require careful dosage and formulation management to avoid secondary deficiencies. The observation of reduced leaf Zn concentration at higher injection doses introduces new challenges in understanding Zn uptake physiology in date palm and paves the way for future research aimed at optimizing Zn application in date palm orchards.

The high‐dose trunk injection treatment with iron (F150) increased the average fruit weight of date palms by approximately 15% compared to the control group. Although this increase did not reach statistical significance (*p* < 0.05), it indicates a positive trend that may hold potential economic relevance for growers. This potential warrants further investigation with larger sample sizes and across diverse agricultural conditions. It is worth noting that this observation differs from the findings of Saleh et al. (2016), who, in a study on date palms, did not observe significant differences in maximum concentration of iron, as well as the highest Brix index and reducing sugars content between various levels of iron sulfate trunk injection (25, 50, and 75 g per tree). This discrepancy may be attributed to differences in formulation (concentration vs. total dose) or genotypic and environmental variations, necessitating further research. Leaf Fe concentration was affected by the different treatments, with the highest recorded for the F150 treatment, consistent with the findings of Saleh (2008), which confirmed the effectiveness of this dosage and application method in supplying the tree's Fe requirements. The present study also showed that Zn_2_ treatment significantly reduced leaf Fe concentration and induced a negative correlation (*r* = −0.297) between Zn and Fe, aligning with multiple previous reports. For instance, Prasad, Prasad et al. (2016) highlighted the classic antagonistic interaction between these two elements, arising from competition during uptake and interference in translocation. Mousavi et al. (2011) similarly reported that Zn application, particularly in calcareous soils with high pH, lowered leaf Fe levels. In pome fruits grown in calcareous soils, foliar Zn application markedly reduced leaf Fe (Rasouli Sadaghiani et al. 2002). This antagonistic interaction may be attributed to competitive absorption sites and translocation interference in calcareous soils with high pH, where excessive Zn uptake can suppress Fe availability and mobilization within plant tissues. The scientific review by Bhat et al. (2024) further emphasized the existence of acquisitional interference between Fe and Zn and its impact on the dynamics of mineral elements in plants. Studies on date palm by Chaâbene et al. (2021) and (Abo‐El‐Saad and Shawir 2024) also observed that changes in the level of one element could lead to an opposite change in the other. In line with this, Abo‐El‐Saad and Shawir (2024) stressed the importance of concurrent management of Fe and Zn to preserve the quality and nutritional value of dates.

In the present study, Fe treatments at 25 and 75 g significantly reduced B content compared with the control. However, increasing the Fe level (F150) led to a rise in B concentration, with the highest B level recorded in root injection treatments, which differed significantly from the control. In a study on pear trees, the combination of Fe and B resulted in higher B concentrations in both leaves and fruits, indicating that Fe may facilitate B uptake and mobility within the tree (Başar and Gürel 2016). Trunk injection of ferrous sulfate effectively corrects Fe deficiency, which can indirectly influence the overall balance of nutrients including B levels by improving tree health and nutrient absorption (Wallace and Wallace 1986). B is an essential element for plants, playing a critical role in various metabolic functions (Marschner 1995). Research on fruit trees has shown that among all nutrients, N, B, and Zn have the greatest impact on fruit set, and the demand for these elements is particularly high during certain phenological stages, such as fruit initiation (Morshedi 2001). Furthermore, results from the present study revealed that Fe application increased leaf Mg and Ca levels in the Fe150 treatment, and raised Cu content in the Fe75 treatment. With higher Fe dosage (F150), an upward trend in K and Ca concentrations in leaves was also observed. These findings are consistent with previous reports indicating increased K, Ca, and Mn levels in peanut plants following Fe fertilization (Zaharieva 1995). However, in the present study, no significant relationship was observed between increased levels of injected Fe and leaf P content in date palm, despite the interaction between Fe and P in plants being documented in numerous studies. For example, FeSO_4_ solution injection may enhance the availability and mobility of other nutrients such as Zn, Mn, and P by lowering the pH of plant sap (Taiz and Zeiger 2002). In a study on apple trees, increasing Fe levels reduced P content (Zhang et al. 2020). Such differences in results may be attributed to variations in plant species, soil conditions, and applied dosages. Fe is the fourth most abundant element in the Earth's crust and, as an essential micronutrient, plays a critical role in activating electron carriers in photosystems I and II and in overall photosynthetic processes (Marschner 1995). This outcome is consistent with literature suggesting that increased Fe levels can support metabolic pathways that promote higher photosynthetic rates and carbohydrate translocation within plant tissues (Mengel and Kirkby 1987), and by improving photosynthesis, not only enhances plant growth but also facilitates the uptake of other elements, particularly micronutrients (Kochian 2000). Fe deficiency directly affects the production of plant pigments such as chlorophyll, carotene, and xanthophyll, with visible symptoms in fruit trees including stunted growth and smaller fruit size (Rout and Sahoo 2015), consistent with the reduced fruit weight observed in some treatments of this study. Soil Fe concentration depends on several factors, including soil acidity, organic matter content, and phosphate fertilizer use, which can influence root uptake. In this context, trunk injection of Fe can supply adequate amounts of this essential element independently of soil pH, which often impedes nutrient uptake and translocation (Fernández‐Escobar et al. 1993). Significant effects of trunk‐injected Fe on enhancing Fe uptake and translocation in date palm (Saleh 2008; Saleh et al. 2016) and increasing plant yield (Peryea and Kammereck 1996) have been reported by various researchers, and the results of the present study further support these positive effects.

In this study, Ca treatments had significant effects on leaf nutrient concentrations. The application of these treatments substantially increased leaf Ca levels while reducing leaf P content. At the lower concentration (Ca_3_), leaf magnesium content also increased compared with the control. These results are in agreement with findings from other studies, including (Danner et al. 2015), which reported increased leaf Ca following Ca application. Ca ions play a crucial role in plant physiological processes, including strengthening the cell wall (Marschner 1995), regulating cell membrane stability, and increasing cell sap concentration. This element also mitigates damage caused by environmental stresses such as heat stress (Shekofteh 2015), drought, and frost. However, Ca ion translocation within plant organs after root uptake can be challenging, and absorption rates are influenced by factors such as sudden increases in temperature and decreases in relative humidity (Marschner 1995). Consequently, plants in warmer regions often experience Ca deficiency. Therefore, maintaining adequate Ca concentrations, especially under stress conditions, is of particular importance. The results of the present study showed that Ca treatments, similar to Fe and Zn treatments, increased date fruit weight in the examined samples. These findings emphasize the vital role of Ca as an essential nutrient for fruit growth and development. Fruit weight, as a key trait, holds high economic importance and directly influences the marketability of the product. Numerous reports have emphasized the existence of a significant positive relationship between fruit yield and traits such as single fruit weight, fruit flesh weight, and fruit diameter (Quispe‐Choque and Huanca‐Alanoca 2023; Rekis et al. 2020). These correlations indicate a close association between fruit quality characteristics and production potential, and they can be utilized in breeding programs and orchard management practices. Moreover, examining nutrient interactions in the soil–plant system provides important insights. Studies on tomato plants have shown that optimal levels of Ca and magnesium can significantly improve growth parameters and yield, suggesting interdependence in the availability of these two nutrients (Aghofack‐Nguemezi et al. 2014). However, this relationship can be complex; for example, excessive magnesium content in soil can compete with other nutrients such as K, Ca, and Mn for the same uptake sites (Panhwar et al. 2020; Zhang et al. 2021). Such antagonism between K and Ca uptake mediated by magnesium has also been observed in the application of magnesium compounds to oil palm (Mohamad 2003). In the present study, similar patterns in elemental correlations were observed (Saleh et al. 2016). Boron exhibited a significant positive correlation with N (0.484), P (0.550), and K (0.668). The addition of boron, together with Fe increased leaf N, K, and P contents. Duncan's multiple range test in this study also revealed a significant negative correlation between Zn and magnesium. Zn is essential for numerous physiological processes, and its deficiency—particularly in tropical soils where availability is often limited—can severely affect plant growth and yield (Jalal et al. 2024). However, it should be noted that excessive Zn levels can also lead to magnesium deficiency in plants. This phenomenon has been observed in rice, where high Zn concentrations inhibit magnesium uptake due to competition for shared transporters in root cells (Fu et al. 2024). Correlations between traits can be exploited for indirect measurement; namely, if two traits are highly correlated, measuring one can allow prediction of the other's status (Wei et al. 2002). Such an approach can be highly useful in optimizing fertilizer use and planning nutrient management strategies for date palm orchards.

While the present study demonstrates the short‐term efficacy of trunk and root injections for improving fruit traits and leaf mineral status in “Mazafati” date palm, the long‐term sustainability of repeated applications warrants careful consideration. Regarding tree health, trunk injection inevitably creates wounds that, if not properly managed, may serve as entry points for pathogens or compromise structural integrity over time (Archer et al. 2022). However, studies on woody species indicate that date palm and similar monocots exhibit efficient compartmentalization of injection wounds, with internal discoloration typically confined to < 10% of the vascular area near the injection site and minimal longitudinal spread (Hauer et al. 2022). To mitigate risks, we followed best practices: disinfecting injection sites with KMnO_4_, sealing wounds with sterile plugs, and limiting injection frequency to once per season. Future work should monitor cumulative wound responses and xylem functionality over multiple years.

Concerning soil chemistry, trunk injection offers a distinct advantage over soil application by delivering nutrients directly into the vascular system, thereby minimizing leaching, fixation, or unintended alteration of rhizosphere chemistry (Archer et al. 2022). Nevertheless, root injection—though used here only for comparative purposes at a single concentration—could theoretically influence localized soil micronutrient pools if applied repeatedly. Long‐term field studies on calcareous soils similar to our experimental site indicate that sustained micronutrient fertilization can gradually modify soil pH, organic matter, and availability of interacting elements (e.g., Zn–Cu antagonism, Fe–P interactions) (Wang et al. 2022). In our case, the low injection volume (320 mL tree^−1^) and targeted delivery likely limit such effects, but periodic soil monitoring is recommended for orchards adopting injection‐based nutrition programs. Finally, nutrient balance remains a critical consideration. Our correlation analyses revealed antagonistic interactions (e.g., Zn–Fe, Zn–Mg), suggesting that repeated high‐dose injections of a single element could inadvertently induce secondary deficiencies. This aligns with the broader principle that micronutrient management should follow a “balanced fertilization” approach, where injection formulations and frequencies are adjusted based on periodic leaf and soil diagnostics. Integrating trunk injection with complementary practices—such as organic amendments to improve soil buffering capacity or rotational use of foliar sprays—may enhance long‐term sustainability while preserving the method's efficiency advantages.

## 5 Study Limitations and Food Safety Considerations

A limitation of the present study is the absence of quantitative residue analysis for injected compounds (ferrous sulfate, zinc aminochelate, calcium aminochelate) in fruit tissues. While trunk injection delivers nutrients directly into the xylem—potentially reducing surface residues compared to foliar applications—future work should include targeted residue monitoring (e.g., via ICP‐MS or HPLC) to verify that fruit concentrations of Fe, Zn, and Ca remain within safe, nutritionally beneficial ranges.

That said, the compounds evaluated in this study have well‐documented safety profiles. Ferrous sulfate is classified as Generally Recognized As Safe (GRAS) by the U.S. FDA and is widely used as a food fortificant (EFSA 2015). Similarly, amino acid‐chelated Zn and Ca are considered low‐risk formulations, as the chelating ligands (e.g., glycine, glutamic acid derivatives) are naturally occurring metabolites that undergo rapid biodegradation (Kurdy and Al‐Dulaimy 2023). Moreover, the low injection volume used here (320 mL tree^−1^) and the dilution effect of transpiration‐driven xylem flow likely minimize the risk of localized accumulation in edible tissues.

Nevertheless, given the direct relevance of date fruits to human nutrition, we strongly recommend that subsequent studies—particularly those aiming for commercial translation—incorporate residue quantification in flesh, skin, and seed compartments, alongside comparison to established maximum residue limits (MRLs) or tolerable upper intake levels (ULs) (WHO 2021). Integrating such analyses will help ensure that nutrient‐enhancement strategies via trunk injection align with both agronomic goals and food safety standards.

Although this study provides valuable insights into nutrient injection effects on “Mazafati” date palm, several limitations warrant consideration when interpreting the findings. First, the experiment was conducted over a single growing season at one orchard location (Azizabad–Bam Research Station). Date palm responses to nutrient management can vary with soil type, climate, and tree age (Marschner 1995); thus, the observed effects may not be fully generalizable to other agroecological zones or cultivars without further validation. Second, the replication level (*n* = 3) and sample size per tree (60 fruits, 30 for lab analysis), while consistent with horticultural field trial standards, may limit statistical power to detect small but biologically meaningful treatment effects, particularly for interactions among multiple elements. Third, the concentration ranges tested for each nutrient were selected based on preliminary data and grower feasibility, but may not represent the true optimum; dose–response curves with finer gradations could refine application recommendations. Fourth, although leaf mineral concentrations were measured, we did not assess physiological mechanisms (e.g., enzyme activity, photosynthetic efficiency, or hormone profiles) that may mediate the observed fruit trait improvements; future work integrating biochemical assays would strengthen causal inference. Fifth, as noted previously, residue analysis in fruit tissues was not conducted, introducing uncertainty regarding food safety implications of repeated injections. Finally, an economic cost–benefit analysis of injection versus conventional fertilization was beyond the scope of this study, yet is critical for grower adoption.

Collectively, these limitations suggest that while our conclusions regarding the potential of trunk injection to improve fruit traits are well‐supported by the data, they should be viewed as preliminary and context‐dependent. The positive trends observed—particularly for Fe and Zn at higher doses—warrant confirmation through multi‐location, multi‐year trials with expanded replication, mechanistic measurements, residue monitoring, and economic evaluation. Until such data are available, we recommend that growers adopt injection‐based nutrition programs cautiously, with on‐farm pilot testing and periodic leaf/soil diagnostics to ensure nutrient balance and product safety.

## 6 Conclusion

Trunk injection of iron, zinc, and calcium effectively enhanced key fruit traits and altered leaf mineral profiles in “Mazafati” date palms. Treatments with higher Fe (150 g L^−1^) and Zn (4 g L^−1^) concentrations yielded the greatest increases in fruit flesh weight, seed weight, and overall fruit mass, demonstrating their potential to improve yield under orchard conditions. Stepwise regression identified fruit flesh weight and leaf phosphorus concentration as the strongest predictors of total fruit weight (*R* ^2^ = 82.1%). While beneficial synergies were observed (e.g., Fe–Mg), antagonistic interactions—particularly Zn‐induced reductions in leaf Fe and Mg—underscore the necessity of balanced nutrient formulations to prevent secondary deficiencies.

In summary, targeted trunk injection of micronutrients offers a viable strategy for enhancing date palm productivity, particularly in calcareous soils where conventional fertilization is inefficient. However, given the single‐season scope and absence of residue and economic analyses in this study, these findings should be interpreted as preliminary. Future research should prioritize multi‐year, multi‐location trials alongside assessments of long‐term tree health, food safety, and cost–benefit feasibility to support sustainable commercial adoption.

## Author Contributions

**Bahman Panahi:** conceptualization, writing – original draft, supervision, project administration. **Ali Salehi Sardoei:** writing – original draft, data curation, software, formal analysis. **Bahareh Damankeshan:** investigation, visualization, data curation. **Mansour Ghorbanpour:** writing – review and editing, methodology, validation, Software.

## Ethics Statement

The authors have nothing to report.

## Consent

The authors have nothing to report.

## Conflicts of Interest

The authors declare no conflicts of interest.

## Data Availability

The data supporting the findings of this study are available from the corresponding author upon reasonable request.
